# Treatment patterns and outcomes of patients with a diagnosis of metastatic pancreatic adenocarcinoma in the United States, 2019–2024

**DOI:** 10.3389/fonc.2026.1844261

**Published:** 2026-07-20

**Authors:** Rupali Fuldeore, Chia Jie Tan, Tomomi Kimura, Pegah Farrokhi, Rachel Yang, Connor Willis, Carl Asche, David Stenehjem

**Affiliations:** 1 Astellas Pharma Inc., Northbrook, IL, United States; 2 Department of Pharmacotherapy, College of Pharmacy, University of Utah, Salt Lake City, UT, United States; 3 Department of Pharmaceutical Care and Health Systems, College of Pharmacy, University of Minnesota, Minneapolis, MN, United States; 4 Pharmacotherapy Outcomes Research Center, University of Utah, Salt Lake City, UT, United States

**Keywords:** FOLFIRINOX, gemcitabine plus nab-paclitaxel, metastatic pancreatic adenocarcinoma, real-world evidence, treatment patterns

## Abstract

**Background:**

Approximately half of patients with pancreatic cancer present with metastatic disease at diagnosis, for whom 5−year survival remains ~3%. This retrospective study assessed treatment patterns, characteristics associated with first-line (1L) regimen selection, and real-world survival and treatment outcomes in US patients with metastatic pancreatic adenocarcinoma (mPAC).

**Methods:**

US adults newly diagnosed with mPAC between 1 January 2019 and 1 August 2024 from the electronic health record-derived Flatiron Health Research Database were included. Treatment patterns across the first three lines of therapy were summarized among patients who initiated systemic therapy within 180 days of metastatic diagnosis. Associations between baseline characteristics and 1L regimen selection (across multiple regimen categories) were evaluated using multinomial logistic regression. Overall survival (OS) and time-to-treatment discontinuation (TTD) were assessed using standard survival methods.

**Results:**

Among 9,439 patients with mPAC, 6,279 initiated 1L systemic therapy. The most common 1L regimens were gemcitabine plus nab-paclitaxel (Gem-Nab; 41.5%) and folinic acid (leucovorin), fluorouracil (FU), irinotecan, and oxaliplatin (FOLFIRINOX) (36.3%). Mean time to 1L initiation after metastatic diagnosis was 26.9 days. Patients receiving 1L FOLFIRINOX were generally younger, more frequently had baseline Eastern Cooperative Oncology Group performance status 0 or 1, more often presented with *de novo* metastatic disease, had higher socioeconomic indicators, and were more likely to be treated in academic centers compared with patients receiving Gem-Nab. Overall, 33.5% of patients did not receive systemic therapy within 180 days of metastatic diagnosis. Median OS was longer among patients treated with FOLFIRINOX than among those treated with Gem-Nab [10.7 vs 7.6 months; adjusted hazard ratio (HR), 0.47; 95% confidence interval (CI), 0.43–0.51; *P* < 0.001]. Median TTD was also longer for FOLFIRINOX compared with Gem-Nab (4.7 vs 3.4 months).

**Conclusions:**

Among treated patients, clinical and sociodemographic factors strongly influenced regimen selection, and FOLFIRINOX use was associated with longer survival and treatment persistence than Gem-Nab; although these findings should be interpreted as associative given the substantial differences in patient characteristics between treatment groups. Conversely, one-third of patients did not initiate systemic therapy within 180 days of metastatic diagnosis, which may reflect unmet need or gaps in access to care.

## 1 Introduction

Pancreatic cancer is the third leading cause of cancer-related death in the United States (US). Its incidence has risen by approximately 1% per year since the late 1990s (1–5). In 2025, there were estimated to be over 67,000 new cases of pancreatic cancer and approximately 52,000 deaths in the US, with mortality increasing by 0.2%–0.3% annually over recent decades (5). Although pancreatic cancer constitutes only 3.3% of all newly diagnosed cancers in the US, it accounts for approximately 8.4% of cancer-related deaths (2). The 5-year relative survival rate for pancreatic cancer remains low at 13.3%, reflecting minimal improvement in long-term survival over the past few decades (1, 2). Pancreatic cancer occurs in both men and women, although incidence is slightly higher in men (15.5 vs 12.3 per 100,000 per year). Nearly 70% of new cases are diagnosed in patients aged ≥ 65 years (2).

Pancreatic ductal adenocarcinoma (PDAC), the predominant histologic subtype of pancreatic cancer, is frequently diagnosed at an advanced or metastatic stage owing to its typically asymptomatic early progression (4, 6, 7). Approximately 50%–55% of patients present with metastatic disease at diagnosis, and the 5-year relative survival rate for metastatic pancreatic cancer is 3.2%, among the lowest for all major cancers (2, 4). This late-stage diagnosis precludes curative treatment and highlights the urgent need for more effective therapies (1–4, 6, 7).

Despite its high mortality, treatment options for metastatic pancreatic adenocarcinoma (mPAC) are limited (1, 3, 4, 6, 8, 9). Current recommended first-line (1L) regimens include the following: folinic acid (leucovorin), fluorouracil (FU), irinotecan, and oxaliplatin (FOLFIRINOX); gemcitabine plus nab-paclitaxel (Gem-Nab); and nanoliposomal irinotecan plus FU, leucovorin, and oxaliplatin (NALIRIFOX) (4, 6, 7, 10, 11). Landmark trials such as ACCORD 11 and NAPOLI-3 have established efficacy benchmarks for these regimens; however, real-world treatment patterns and survival outcomes may differ from those observed in clinical trials (8, 12, 13). Although immunotherapy and targeted therapies, such as entrectinib or larotrectinib for neurotrophic tyrosine receptor kinase gene fusions, and pembrolizumab for tumors exhibiting microsatellite instability-high, mismatch repair deficiency, or high tumor mutational burden, are approved for select subsets of patients with PDAC, these alterations are rare, each found in fewer than 2% of patients (14, 15). As a result, the impact of real-world use of these targeted therapies is limited (14, 15).

Real-world evidence in mPAC is essential because clinical trial populations often differ from those encountered in routine practice, and gaps remain in understanding treatment patterns and outcomes outside controlled settings (16–19). Multiple factors including patient demographics, Eastern Cooperative Oncology Group performance status (ECOG PS), comorbidities, socioeconomic status, and practice setting influence treatment selection and outcomes for patients with mPAC; however, these real-world determinants and their impact are not well characterized in routine clinical practice (1, 6, 10, 11, 20, 21). Although multiple guideline-recommended regimens are available, real-world survival for patients with mPAC is substantially shorter than clinical trial benchmarks (8, 9, 13, 19, 22). Comparative effectiveness of 1L regimens in unselected, real-world populations remains unclear, with limited data on outcomes such as overall survival (OS), time-to-treatment discontinuation (TTD), and time to next treatment (20, 22, 23).

Addressing these gaps is critical for informing clinical decision-making, identifying undertreated populations, and optimizing the use and positioning of emerging therapies (16–18, 22). Notably, a substantial proportion of patients with mPAC do not receive systemic anticancer treatment, often due to poor ECOG PS and comorbidities, further highlighting disparities in care (18, 24, 25). This retrospective study aimed to address gaps in real-world evidence by characterizing treatment patterns up to three lines of therapy, identifying factors associated with receipt of 1L regimens, and evaluating effectiveness outcomes including OS, TTD, and time to next treatment using standard survival analysis methods in patients with mPAC.

## 2 Materials and methods

### 2.1 Study design

This study was conducted as a retrospective cohort analysis that used deidentified, electronic health record (EHR)-derived data on mPAC from the Flatiron Health Research Database. The database included information from both community and academic cancer centers across the US and contained structured and unstructured EHR data. Unstructured data were extracted using a combination of human review, technology-assisted processes, and natural language processing tools. All data were maintained in a deidentified format. The study period extended from 1 January 2019 through 28 February 2025. The date of metastatic diagnosis was defined as the index date. Observation windows for baseline comorbidities were defined as 12 months before the index date through 1 month after the index date; other time-varying characteristics were defined as 30 days before the index date through 7 days after the index date.

### 2.2 Study population and eligibility criteria

The study population consisted of adult patients (aged ≥ 18 years) with a diagnosis of mPAC in the US between 1 January 2019 and 1 August 2024 identified from the Flatiron Health Research Database. Patients were identified using International Classification of Diseases, Ninth Revision, Clinical Modification (ICD-9-CM) code 157.x and ICD, Tenth Revision, Clinical Modification (ICD-10-CM) code C25.x; diagnoses were confirmed through review of clinical notes and pathology reports to verify adenocarcinoma histology and metastatic disease. All patients were required to have ≥ 2 documented clinical visits on separate dates after diagnosis of metastatic disease. Patients were excluded if they had more than one primary cancer during the study period or if they received 1L treatment for mPAC in a clinical trial setting.

Patients were followed until the end of the study period, death, or last documented visit. First-line treatment was assessed within 180 days of metastatic diagnosis.

### 2.3 Outcomes

Outcomes included the distribution of patients receiving each treatment regimen across the first three lines of therapy; the proportion of patients who did not receive any systemic treatment within the first 180 days from index date; the odds of receiving a specific 1L regimen according to patient characteristics; and the time from diagnosis of metastatic disease to initiation of 1L therapy. Regimens evaluated included FOLFIRINOX, Gem-Nab, NALIRIFOX, and other systemic therapies as documented in the Flatiron Health Research Database. Briefly, regimens included drugs indicated for metastatic pancreatic cancer that were started within 28 days. First-line treatments were summarized according to baseline ECOG PS, which was also included as a covariate in analyses assessing treatment patterns. Treatment pathways were assessed in relation to disease staging at initial presentation (*de novo* metastatic or progressed from nonmetastatic disease, defined here as initial early−stage or locally advanced disease with subsequent metastatic recurrence).

Effectiveness endpoints included OS, defined as the interval between the date of metastatic diagnosis and death, and TTD, defined as the interval between the first and last administration dates of 1L therapy, in which the last administration was defined by a ≥ 56-day gap without further treatment. Real−world progression data were not available in the database; therefore, real−world progression−free survival could not be estimated. TTD was used as a practical real−world measure of treatment duration and persistence rather than as a direct surrogate for disease progression (26).

The study was approved by the University of Utah Institutional Review Board (IRB_00182082; approval date: 4 December 2024), with a waiver of informed consent due to the use of deidentified data.

### 2.4 Statistical analysis

All eligible patients in the Flatiron database were included, providing sufficient sample size to support comparative effectiveness analyses with 80% power at a two-sided alpha = 0.05. Treatment patterns were summarized descriptively for ≤ 3 lines of therapy from the time of mPAC diagnosis. Associations between patient characteristics and selection of 1L regimen were evaluated using logistic regression for (i) 1L regimen categorized as the two regimens with at least 20% patients, namely, FOLFIRINOX and Gem-Nab, and (ii) no treatment versus receipt of 1L therapy. Unadjusted associations were evaluated using bivariate logistic and Cox regression models. Adjusted associations were evaluated using multivariable regression models to generate adjusted estimates. Covariates were evaluated in bivariate models and retained in final multivariable models based on prespecified clinical/statistical criteria; adjusted estimates are presented for covariates retained in the final model. Covariates considered included age, sex, race and ethnicity, smoking history, insurance type, practice setting, socioeconomic status, baseline ECOG PS, disease stage at presentation, and baseline comorbidities. Kaplan–Meier methods were used for OS and TTD, with Cox proportional hazards models used for comparative analyses when the proportional hazards assumptions were met. Censoring was performed at the date of last documented activity, initiation of a clinical trial drug, or end of the study period. The models were intended to assess the main effects of clinically relevant variables; interaction terms and nonlinear effects were not evaluated, consistent with the observational and hypothesis-generating nature of the analysis.

A complete case analysis approach was used for all analyses except for the assessment of OS between patients who received no treatment, FOLFIRINOX, and Gem-Nab. As a result, the analytic cohorts for multivariable models represented subsets of treated patients with complete baseline covariate data and were smaller than the overall population receiving 1L therapy. Baseline ECOG PS was incompletely documented, with missing values disproportionately concentrated among patients who did not initiate any systemic 1L therapy. To address this, baseline ECOG PS missing values were estimated for OS evaluation using multiple imputation by chained equations for patients with complete data for all other analytic variables. ECOG PS (0–4) values were imputed using an ordinal logistic regression model based on all other analytic covariates, generating 20 imputed datasets; the imputed values were then recategorized as 0–1 and 2–4 to assess the associations with outcomes of interest.

OS was measured from the date of metastatic diagnosis for all patients to ensure a consistent time origin across treatment groups, given the variation in treatment initiation timing in routine clinical practice. However, comparisons between treated and untreated patients may be subject to immortal time bias, and resulting survival estimates should be interpreted cautiously.

## 3 Results

### 3.1 Patient characteristics

As of the data cutoff date (28 February 2025), 9,439 patients with mPAC were included in the analysis (**Supplementary Table 1**). The mean [standard deviation (SD)] age at metastatic diagnosis was 69.2 (9.8) years, and most patients (69.5%) were aged ≥ 65 years. Slightly more than half of the cohort were men (52.8%), and 50.7% were non-Hispanic White. Most patients had commercial health insurance (41.7%) or Medicare (43.2%), and the majority were treated in community practice settings (70.6%).

At baseline, 50.6% of patients had a reported ECOG PS of 0 or 1. The majority (63.5%) presented with *de novo* metastatic disease, whereas 27.5% presented with an initial diagnosis of locally advanced disease; 9.1% had unreported staging. Patient characteristics were further stratified by 1L treatment, which included no treatment, FOLFIRINOX, other FU-based regimens, Gem-Nab, other gemcitabine-based regimens, and other regimens.

### 3.2 Treatment patterns

#### 3.2.1 Treatment regimens by line of therapy

Of the 9,439 patients with mPAC included, 33.5% did not receive any systemic anticancer treatment within 180 days of metastatic diagnosis. For patients who received treatment, the most common 1L regimens were Gem-Nab (41.5%) and FOLFIRINOX (36.3%), with smaller proportions receiving NALIRIFOX and variants (2.9%); folinic acid, FU, and oxaliplatin (FOLFOX) or folinic acid, FU, and irinotecan (FOLFIRI) (5.3%); FU monotherapy (1.6%); other FU-based regimens (3.3%); gemcitabine monotherapy (5.9%); other gemcitabine-based regimens (1.5%); or other regimens (1.8%; **Table 1**).

**Table 1:** Treatment regimens by line of therapy.

Treatment regimen, *n* (%)	1L (*n* = 6,279)	2L (*n* = 2,460)	3L (*n* = 814)
Gem-Nab	2,605 (41.5)	1,014 (41.2)	126 (15.5)
FOLFIRINOX^a^	2,277 (36.3)	182 (7.4)	41 (5.0)
Gem monotherapy	371 (5.9)	77 (3.1)	20 (2.5)
FOLFOX/FOLFIRI	330 (5.3)	200 (8.1)	137 (16.8)
Other FU-based regimens	207 (3.3)	124 (5.0)	40 (4.9)
NALIRIFOX and variants	185 (2.9)	450 (18.3)	206 (25.3)
Others^b^	111 (1.8)	138 (5.6)	80 (9.8)
FU monotherapy	99 (1.6)	91 (3.7)	39 (4.8)
Other Gem-based regimens	94 (1.5)	99 (4.0)	65 (8.0)
Clinical trials^c^	0	85 (3.5)	60 (7.4)

^a^ Regimen included modified FOLFIRINOX.

^b^ Treatment regimens used by < 20% of patients were collectively grouped as “Others.”

^c^ Any treatment regimen that included an investigational agent.

1L, first-line; 2L, second-line; 3L, third-line; FOLFIRI, folinic acid, FU, irinotecan; FOLFIRINOX, folinic acid (leucovorin), FU, irinotecan, oxaliplatin; FOLFOX, folinic acid, FU, oxaliplatin; FU, fluorouracil; Gem, gemcitabine; Gem-Nab, gemcitabine plus nab-paclitaxel; NALIRIFOX, nanoliposomal irinotecan plus FU, leucovorin, and oxaliplatin.

The mean (SD) time from metastatic diagnosis to initiation of 1L therapy was 26.9 days (26.1). Compared with 1L treatment patterns, the use of FOLFIRINOX decreased in second-line and third-line settings, while Gem-Nab was more commonly used than FOLFIRINOX, and NALIRIFOX use increased in later lines.

#### 3.2.2 Treatment regimens by line of therapy and disease stage

Within the subgroup of patients with an initial diagnosis of early or locally advanced disease, 35.6% (*n*/*n* = 923/2,593) did not receive 1L treatment compared with 31.0% of patients (*n*/*n* = 1,859/5,991) presenting with *de novo* metastatic disease. The mean (SD) time from metastatic diagnosis to first dose of 1L therapy was 24.0 (30.1) days for patients with early or locally advanced disease at initial presentation and 28.4 (23.4) days for patients with *de novo* metastatic disease.

Treatment selection also varied by disease stage at presentation. Gem-Nab (47.7%) was the most frequently used 1L regimen for patients with an initial diagnosis of early stage or locally advanced disease, whereas FOLFIRINOX was most frequently used in patients with *de novo* metastatic disease (45.7%; **Table 2**).

**Table 2:** Treatment regimens by line of therapy and disease stage.^a^

Treatment regimen, *n* (%)	1L (*n* = 5,802)	2L (*n* = 2,291)	3L (*n* = 768)
Early-stage/locally advanced disease at initial presentation	*n* = 1,670	*n* = 723	*n* = 246
Gem-Nab	796 (47.7)	204 (28.2)	41 (16.7)
FOLFIRINOX^b^	298 (17.8)	37 (5.1)	10 (4.1)
Other FU-based regimens	136 (8.1)	52 (7.2)	9 (3.7)
FOLFOX/FOLFIRI	109 (6.5)	71 (9.8)	43 (17.5)
NALIRIFOX and variants	104 (6.2)	200 (27.7)	54 (22.0)
Gem monotherapy	101 (6.0)	19 (2.6)	12 (4.9)
Others^c^	52 (3.1)	52 (7.2)	25 (10.2)
FU monotherapy	45 (2.7)	30 (4.1)	10 (4.1)
Other Gem-based regimens	29 (1.7)	31 (4.3)	24 (9.8)
Clinical trials^d^	0	27 (3.7)	18 (7.3)
*De novo* metastatic disease at initial presentation	*n* = 4,132	*n* = 1,568	*n* = 522
FOLFIRINOX^b^	1,887 (45.7)	136 (8.7)	30 (5.7)
Gem-Nab	1,586 (38.4)	769 (49.0)	76 (14.6)
Gem monotherapy	240 (5.8)	54 (3.4)	8 (1.5)
FOLFOX/FOLFIRI	190 (4.6)	118 (7.5)	87 (16.7)
Other Gem-based regimens	54 (1.3)	55 (3.5)	36 (6.9)
Other FU-based regimens	50 (1.2)	59 (3.8)	26 (5.0)
FU monotherapy	45 (1.1)	52 (3.3)	26 (5.0)
NALIRIFOX and variants	41 (1.0)	202 (12.9)	139 (26.6)
Others^c^	39 (0.9)	77 (4.9)	52 (10.0)
Clinical trials^d^	0	46 (2.9)	42 (8.0)

^a^ Patients with missing disease staging at initial presentation were excluded from analyses stratified by stage.

^b^ Regimen included modified FOLFIRINOX.

^c^ “Others” included treatment regimens that did not consist of FU−based or gemcitabine−based therapies.

^d^ Any treatment regimen that included an investigational agent.

1L, first-line; 2L, second-line; 3L, third-line; FOLFIRI, folinic acid, FU, irinotecan; FOLFIRINOX, folinic acid (leucovorin), FU, irinotecan, oxaliplatin; FOLFOX, folinic acid, FU, oxaliplatin; FU, fluorouracil; Gem, gemcitabine; Gem-Nab, gemcitabine plus nab-paclitaxel; NALIRIFOX, nanoliposomal irinotecan plus FU, leucovorin, and oxaliplatin.

#### 3.2.3 Treatment regimens by line of therapy and ECOG PS

In patients with a baseline ECOG PS of 0 or 1, 3.9% (*n*/*n* = 184/4,779) did not receive 1L treatment, compared with 4.9% of patients (*n*/*n* = 49/1,006) with a baseline ECOG PS of 2, 3, or 4. The mean (SD) time from metastatic diagnosis to first dose of 1L therapy was 26.5 days (25.6) for patients with an ECOG PS of 0 or 1 and 29.5 days (28.3) for patients with an ECOG PS of 2, 3, or 4.

Gem-Nab was the most frequent 1L treatment regimen across both ECOG PS groups, accounting for 40.8% of patients with a baseline ECOG PS of 0 or 1 and 47.4% for patients with a baseline ECOG PS of 2, 3, or 4 (**Table 3**). Notably, 38.7% of the overall cohort did not have a baseline ECOG PS value documented. Among patients who did not initiate any 1L systemic therapy, 92.6% (*n*/*n* = 2,927/3,160) lacked documented baseline ECOG PS.

**Table 3:** Treatment patterns by line of therapy and ECOG PS.^a^

Treatment regimen, *n* (%)	1L (*n* = 5,552)	2L (*n* = 2,189)	3L (*n* = 736)
Baseline ECOG PS 0/1	*n* = 4,595	*n* = 1,979	*n* = 675
Gem-Nab	1,877 (40.8)	816 (41.2)	105 (15.6)
FOLFIRINOX^b^	1,800 (39.2)	142 (7.2)	37 (5.5)
FOLFOX/FOLFIRI	201 (4.4)	157 (7.9)	104 (15.4)
Gem monotherapy	193 (4.2)	56 (2.8)	16 (2.4)
NALIRIFOX and variants	151 (3.3)	368 (18.6)	166 (24.6)
Other FU-based regimens	148 (3.2)	95 (4.8)	33 (4.9)
FU monotherapy	80 (1.7)	74 (3.7)	32 (4.7)
Others^c^	75 (1.6)	112 (5.7)	73 (10.8)
Other Gem-based regimens	70 (1.5)	88 (4.4)	56 (8.3)
Clinical trials^d^	0	71 (3.6)	53 (7.9)
Baseline ECOG PS 2/3/4	*n* = 957	*n* = 210	*n* = 61
Gem-Nab	454 (47.4)	59 (28.1)	8 (13.1)
FOLFIRINOX^b^	180 (18.8)	22 (10.5)	2 (3.3)
Gem monotherapy	139 (14.5)	14 (6.7)	2 (3.3)
FOLFOX/FOLFIRI	72 (7.5)	25 (11.9)	16 (26.2)
Other FU-based regimens	34 (3.6)	16 (7.6)	2 (3.3)
NALIRIFOX and variants	27 (2.8)	43 (20.5)	16 (26.2)
Others^c^	24 (2.5)	12 (5.7)	3 (4.9)
Other Gem-based regimens	18 (1.9)	4 (1.9)	5 (8.2)
FU monotherapy	9 (0.9)	10 (4.8)	3 (4.9)
Clinical trials^d^	0	5 (2.4%)	4 (6.6)

^a^ Patients with missing ECOG PS at initial presentation were excluded from analyses stratified by ECOG PS.

^b^ Regimen included modified FOLFIRINOX.

^c^ “Others” included treatment regimens that did not consist of FU−based or gemcitabine−based therapies.

^d^ Any treatment regimen that included an investigational agent.

1L, first-line; 2L, second-line; 3L, third-line; ECOG PS, Eastern Cooperative Oncology Group performance status; FOLFIRI, folinic acid, FU, irinotecan; FOLFIRINOX, folinic acid (leucovorin), FU, irinotecan, oxaliplatin; FOLFOX, folinic acid, FU, oxaliplatin; FU, fluorouracil; Gem, gemcitabine; Gem-Nab, gemcitabine plus nab-paclitaxel; NALIRIFOX, nanoliposomal irinotecan plus FU, leucovorin, and oxaliplatin.

#### 3.2.4 Treatment regimens by line of therapy and practice site

By practice setting, 29.2% of patients (*n*/*n* = 1,946/6,665) managed in community centers did not receive 1L treatment compared with 43.8% of patients (*n*/*n* = 1,214/2,774) in academic centers. The mean (SD) time from metastatic diagnosis to first dose of 1L therapy was 26.5 days (25.3) for patients managed in community centers and 27.9 days (28.2) for those managed in academic centers.

Treatment patterns also differed by practice setting. Gem-Nab (43.0%) was the most common 1L regimen in patients managed in community centers, whereas FOLFIRINOX (40.0%) was the most common in those managed in academic centers (**Table 4**).

**Table 4:** Treatment patterns by line of therapy and practice site.

Treatment regimen, *n* (%)	1L (*n* = 6,279)	2L (*n* = 2,460)	3L (*n* = 814)
Treated in community center	n = 4,719	n = 1,777	n = 564
Gem-Nab	2,031 (43.0)	722 (40.6)	87 (15.4)
FOLFIRINOX^a^	1,653 (35.0)	139 (7.8)	28 (5.0)
Gem monotherapy	279 (5.9)	56 (3.2)	15 (2.7)
FOLFOX/FOLFIRI	250 (5.3)	147 (8.3)	90 (16.0)
Other FU-based regimens	148 (3.1)	92 (5.2)	28 (5.0)
NALIRIFOX and variants	142 (3.0)	358 (20.1)	152 (27.0)
Others^b^	82 (1.7)	94 (5.3)	62 (11.0)
FU monotherapy	73 (1.5)	64 (3.6)	33 (5.9)
Other Gem-based regimens	61 (1.3)	72 (4.1)	39 (6.9)
Clinical trials^c^	0	33 (1.9)	30 (5.3)
Treated in academic center	*n* = 1,560	*n* = 683	
FOLFIRINOX^a^	624 (40.0)	43 (6.3)	13 (5.2)
Gem-Nab	574 (36.8)	292 (42.8)	39 (15.6)
Gem monotherapy	92 (5.9)	21 (3.1)	5 (2.0)
FOLFOX/FOLFIRI	80 (5.1)	53 (7.8)	47 (18.8)
Other FU-based regimens	59 (3.8)	32 (4.7)	12 (4.8)
NALIRIFOX and variants	43 (2.8)	92 (13.5)	54 (21.6)
Other Gem-based regimens	33 (2.1)	27 (4.0)	26 (10.4)
Others^b^	29 (1.9)	44 (6.4)	18 (7.2)
FU monotherapy	26 (1.7)	27 (4.0)	6 (2.4)
Clinical trials^c^	0	52 (7.6)	30 (12.0)

^a^ Regimen included modified FOLFIRINOX.

^b^ “Others” included treatment regimens that did not consist of FU−based or gemcitabine−based therapies.

^c^ Any treatment regimen that included an investigational agent.

1L, first-line; 2L, second-line; 3L, third-line; FOLFIRI, folinic acid, FU, irinotecan; FOLFIRINOX, folinic acid (leucovorin), FU, irinotecan, oxaliplatin; FOLFOX, folinic acid, FU, oxaliplatin; FU, fluorouracil; Gem, gemcitabine; Gem-Nab, gemcitabine plus nab-paclitaxel; NALIRIFOX, nanoliposomal irinotecan plus FU, leucovorin, and oxaliplatin.

### 3.3 Factors associated with receipt of 1L FOLFIRINOX compared with 1L Gem-Nab

Several patient characteristics were independently associated with receipt of FOLFIRINOX as 1L treatment (**Table 5**). Increasing age was associated with lower odds of receiving FOLFIRINOX [OR = 0.92; 95% confidence interval (CI): 0.91–0.93; *P* < 0.001]. Treatment setting also influenced regimen selection; patients treated in community centers had lower odds of receiving FOLFIRINOX compared with those treated in academic centers (OR = 0.79; 95% CI: 0.65–0.96; *p* = 0.02). Socioeconomic status demonstrated a positive association with FOLFIRINOX use, with the strongest effect observed among patients residing in areas with the highest socioeconomic status (Quintile 5: OR = 1.72; 95% CI: 1.27–2.34; *p* < 0.001).

**Table 5:** Association between patient characteristics and receipt of 1L FOLFIRINOX versus 1L Gem-Nab (reference) (*n* = 2,601).

Variable	Unadjusted analysis		Adjusted analysis	
	OR (95% CI)	*P*-value	OR (95% CI)	*P*-value
**Age at diagnosis**	0.93 (0.92–0.94)	< 0.001	0.92 (0.91–0.93)	< 0.001
**Sex**		0.25		
Male	Reference			
Female	1.1 (0.94–1.28)			
**Race and ethnicity**		0.08		
Non-Hispanic White	Reference			
Non-Hispanic Black	0.95 (0.74–1.21)			
Asian	1.55 (0.90–2.66)			
Hispanic or Latino	0.99 (0.75–1.30)			
Others	0.68 (0.48–0.95)			
**History of smoking**		0.89		
Yes (current and ex-smoker)	Reference			
No	0.99 (0.85–1.15)			
**Insurance coverage**		< 0.001		
Commercial health plan	Reference		Reference	
Medicare	0.53 (0.45–0.63)		0.87 (0.72–1.06)	
Medicaid	0.86 (0.58–1.30)		0.63 (0.40–1.00)	
Others	1.1 (0.75–1.60)		0.71 (0.46–1.10)	
Self-pay/no documented payer	1.28 (0.76–2.15)		1.1 (0.61–1.97)	
**Practice setting**		< 0.001		0.02
Academic	Reference		Reference	
Community	0.72 (0.61–0.85)		0.79 (0.65–0.96)	
**Socioeconomic status**		0.005		< 0.001
Quintile 1 (lowest)	Reference		Reference	
Quintile 2	0.9 (0.68–1.18)		1.04 (0.77–1.42)	
Quintile 3	1.23 (0.95–1.95)		1.44 (1.08–1.93)	
Quintile 4	1.3 (1.01–1.66)		1.66 (1.25–2.21)	
Quintile 5 (highest)	1.34 (1.03–1.74)		1.72 (1.27–2.34)	
**Baseline ECOG PS**		< 0.001		< 0.001
0/1	Reference		Reference	
2/3/4	0.43 (0.34–0.54)		0.38 (0.30–0.49)	
**Disease staging at initial presentation**		< 0.001		< 0.001
Early stage/locally advanced	Reference		Reference	
* De novo* metastatic disease	3.36 (2.78–4.06)		4.39 (3.56–5.42)	
Baseline comorbidities				
Type 2 diabetes mellitus	0.97 (0.78–1.19)	0.76		
Hypertension	0.79 (0.66–0.94)	0.008	0.81 (0.66–0.99)	0.04
Heart failure	1.11 (0.59–2.08)	0.75		
Immunocompromised conditions	1.67 (0.61–4.52)	0.32		
**Baseline hepatic impairment**	0.85 (0.71–1.01)	0.06		
**Baseline renal impairment**	0.34 (0.17–0.67)	0.002	0.42 (0.20–0.89)	0.02

CI, confidence interval; ECOG PS, Eastern Cooperative Oncology Group performance status; FOLFIRINOX, folinic acid (leucovorin), FU, irinotecan, oxaliplatin; FU, fluorouracil; Gem-Nab, gemcitabine plus nab-paclitaxel; OR, odds ratio.

Baseline performance status was also significantly associated with regimen selection; patients with an ECOG of 2, 3, or 4 were substantially less likely to receive FOLFIRINOX compared with those with an ECOG of 0 or 1 (OR = 0.38; 95% CI: 0.30–0.49; *p* < 0.001). Disease staging at presentation further influenced regimen selection; patients with *de novo* metastatic disease had markedly higher odds of receiving FOLFIRINOX than those initially presenting with early−stage or locally advanced disease (OR = 4.39; 95% CI: 3.56–5.42; *p* < 0.001).

Among comorbidities, hypertension (OR = 0.81; 95% CI: 0.66–0.99; *p* = 0.04) and baseline renal impairment (OR = 0.42; 95% CI: 0.20–0.89; *p* = 0.02) were associated with lower odds of receiving FOLFIRINOX. Insurance type was not independently associated with regimen selection in the adjusted model. No other demographic or clinical factors demonstrated significant associations after adjustment.

### 3.4 Survival and treatment outcomes for 1L FOLFIRINOX compared with 1L Gem-Nab

**Figure 1:**
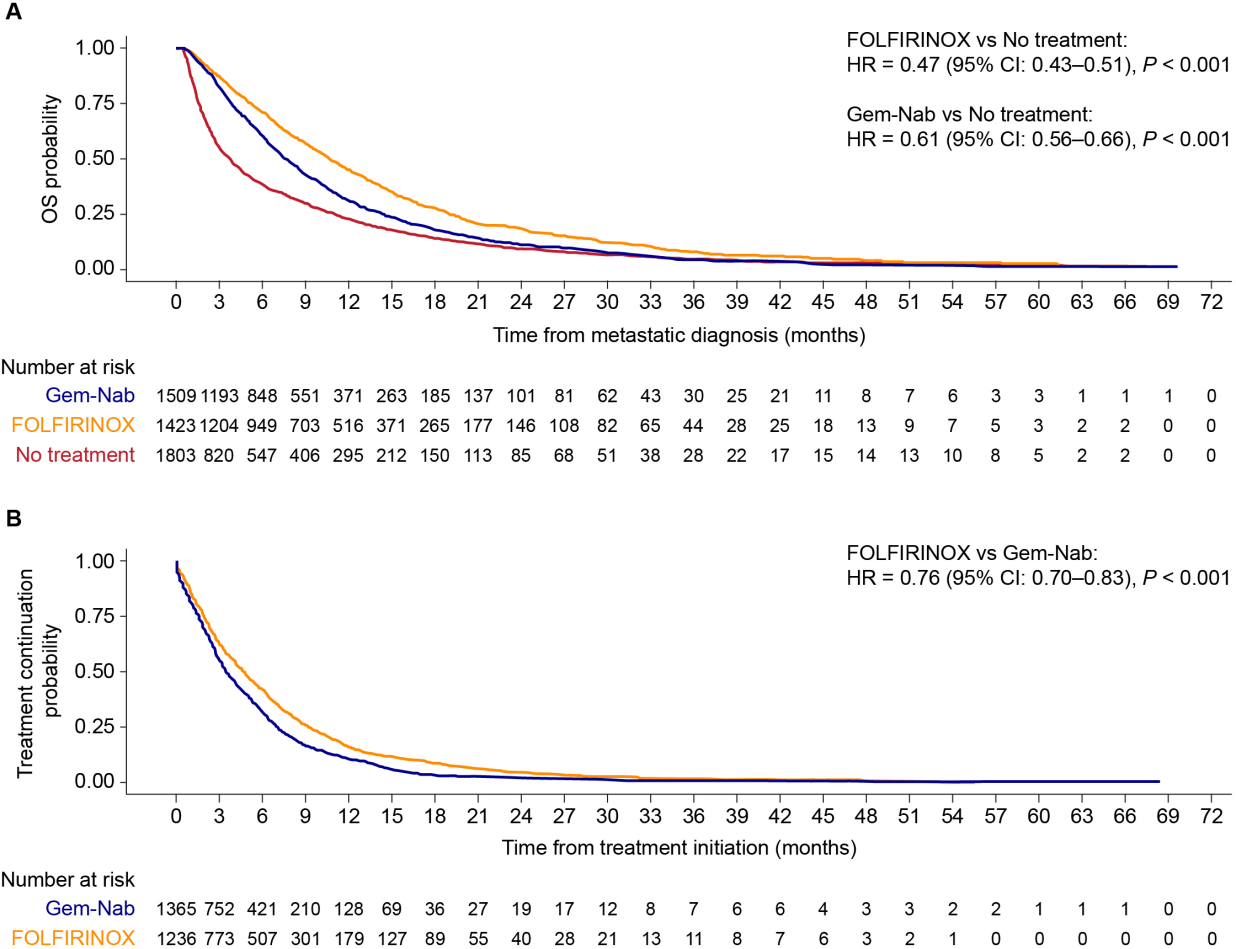
**(A)** OS from metastatic diagnosis and **(B)** TTD from initiation of 1L therapy, by 1L regimen. 1L, first-line; CI, confidence interval; FOLFIRINOX, folinic acid (leucovorin), FU, irinotecan, oxaliplatin; FU, fluorouracil; HR, hazard ratio; Gem-Nab, gemcitabine plus nab-paclitaxel; OS, overall survival; TTD, time-to-treatment discontinuation.

Kaplan–Meier curves demonstrated clear separation in OS and TTD between treatment groups (**Figures 1A, B**). The OS curves (**Figure 1A**) showed consistent separation between treatment groups across most of the follow-up period, with patients treated with FOLFIRINOX generally exhibiting higher survival probabilities than those treated with Gem-Nab. Similar patterns were observed in TTD curves (**Figure 1B**), indicating greater treatment persistence with FOLFIRINOX. These visual patterns were consistent with longer OS and greater on-treatment duration observed for FOLFIRINOX in the multivariable analyses.

A total of 4,735 patients were included in the multivariable OS analysis after excluding patients with incomplete covariate data for variables not addressed through imputation (median follow-up, 4.3 months). The median OS differed markedly by treatment group; patients who did not receive systemic therapy had the shortest survival (3.7 months; 95% CI: 3.4–4.0), followed by patients receiving Gem−Nab (7.6 months; 95% CI: 7.2–8.2), while those treated with FOLFIRINOX experienced the longest survival (10.7 months; 95% CI: 10.0–11.3) (**Table 6**). Proportional hazards assumptions were met for the OS model. After adjustment for baseline characteristics, both Gem−Nab [adjusted hazard ratio (HR) = 0.61; 95% CI: 0.56–0.66; *p* < 0.001] and FOLFIRINOX (adjusted HR = 0.47; 95% CI: 0.43–0.51; *p* < 0.001) were associated with significantly lower mortality risk compared with no treatment.

**Table 6:** OS and TTD in multivariable regression analyses (OS: no treatment, 1L Gem-Nab, and 1L FOLFIRINOX; TTD: 1L FOLFIRINOX versus 1L Gem-Nab).^a^

	n	Events, *n* (%)	Median in months (95% CI)	Unadjusted analysis		Adjusted analysis	
				HR (95% CI)	*P*-value	HR (95% CI)	*P*-value
**OS (in months)** ^b^					< 0.001		< 0.001
No treatment	1,803	1,344 (74.5)	3.7 (3.4–4.0)	Reference		Reference	
Gem-Nab	1,509	1,231 (81.6)	7.6 (7.2–8.2)	0.70 (0.65–0.76)		0.61 (0.56–0.66)	
FOLFIRINOX^c^	1,438	1,089 (75.7)	10.7 (10.0–11.3)	0.54 (0.50–0.58)		0.47 (0.43–0.51)	
**TTD (in months)** ^d^					< 0.001		< 0.001
Gem-Nab	1,365	1,310 (96.0)	3.4 (3.2–3.8)	Reference		Reference	
FOLFIRINOX^c^	1,236	1,167 (94.4)	4.7 (4.3–5.0)	0.78 (0.72–0.84)		0.76 (0.70–0.83)	

^a^ Time zero differed by endpoint: OS was measured from metastatic diagnosis (index date); TTD was measured from 1L treatment initiation (first administration date).

^b^ The multivariable OS model included *n* = 4,735 patients after excluding those with incomplete covariate data for variables not addressed through imputation; group counts shown are descriptive totals.

^c^ Regimen included modified FOLFIRINOX.

^d^ For TTD, discontinuation was defined as a ≥ 56−day gap without administration of the 1L regimen after the last administration date.

1L, first-line; CI, confidence interval, FOLFIRINOX, folinic acid (leucovorin), FU, irinotecan, oxaliplatin; FU, fluorouracil; Gem-Nab, gemcitabine plus nab-paclitaxel; HR, hazard ratio; OS, overall survival; TTD, time-to-treatment discontinuation.

For TTD, 2,601 patients were included in the analysis (median follow−up, 7.0 months). Proportional hazards assumptions were also met for the TTD model. The median TTD was 3.4 months (95% CI: 3.2–3.8) for Gem−Nab and 4.7 months (95% CI: 4.3–5.0) for FOLFIRINOX. In the adjusted model, FOLFIRINOX was associated with a significantly lower hazard of treatment discontinuation compared with Gem−Nab (adjusted HR = 0.76; 95% CI: 0.70–0.83; *p* < 0.001).

## 4 Discussion

In this large, real-world cohort of patients with mPAC, we observed substantial heterogeneity in 1L regimen selection, with approximately one-third of patients receiving no systemic anticancer treatment within 180 days of metastatic diagnosis. Importantly, patients who did not initiate systemic therapy may reflect a heterogeneous group with multiple potential underlying considerations, including factors such as poor functional status, early mortality, preference for hospice- or supportive-focused care, or incomplete treatment capture in real-world EHR data. Although lack of treatment initiation may reflect unmet need or gaps in access for some patients, these findings cannot be directly attributed to a single cause and should not be uniformly interpreted as suboptimal care. This observation is consistent with prior US and international studies reporting that a substantial proportion of patients with advanced pancreatic cancer do not receive systemic anticancer therapy, often due to poor ECOG PS, comorbidities, or advanced age (19, 20, 24).

Consistent with prior evidence, patient factors strongly influenced regimen selection, with treatment options in this cohort largely comprising cytotoxic chemotherapy regimens. In our analysis, younger age, lower ECOG PS, *de novo* metastatic disease, higher socioeconomic status, and treatment in academic centers were independently associated with increased likelihood of receiving FOLFIRINOX as 1L therapy. These determinants mirror the broader literature, demonstrating that patient fitness, sociodemographic factors, and access to specialized care shape systemic treatment decisions in pancreatic cancer (11, 19, 20, 25, 27–29). Although descriptive differences in insurance type were observed across treatment groups, insurance type was not independently associated with 1L regimen selection in adjusted analyses. In contrast, socioeconomic status, treatment setting, and select comorbidities remained significant determinants of regimen selection in adjusted analyses. These disparities are consistent with prior reports showing that lower socioeconomic status and treatment in nonacademic or minority-serving centers are associated with lower rates of guideline-concordant care and poorer outcomes (27, 30). In our cohort, Gem−Nab was more frequently used in the 1L setting among patients with early or locally advanced disease that progressed to metastatic disease compared with those with *de novo* metastatic disease, which may reflect real−world treatment considerations related to tolerability, comorbidity burden, and feasibility (10, 31). In the 1L setting, treatment patterns differed by practice setting, with Gem−Nab more commonly used in community centers and FOLFIRINOX more commonly used in academic centers. This pattern may reflect practical considerations in routine care, including differences in cost drivers across regimens (e.g., higher chemotherapy drug costs for Gem−Nab versus higher chemotherapy administration and supportive care costs, such as granulocyte colony−stimulating factor for FOLFIRINOX-based regimens), which may influence regimen feasibility in real-world settings (32, 33). These potential contributors could not be directly evaluated in this dataset. Further research is needed to better distinguish patient−level, provider−level, and system−level contributors to these observed differences. Practice setting should therefore be interpreted as a marker of care delivery context rather than a causal determinant of treatment selection or outcomes.

In our cohort, treatment with FOLFIRINOX was associated with longer OS than Gem-Nab, with a median OS of 10.7 and 7.6 months, respectively. This difference is clinically meaningful and aligns with trends observed in pivotal trials, while the shorter median OS observed here compared with clinical trial populations may reflect differences in patient selection and the broader, more heterogeneous populations captured in real-world data (8, 23). Real-world studies have similarly reported longer survival with FOLFIRINOX than with Gem-based regimens (19, 29), and the magnitude of benefit in our analysis is consistent with observational data showing a higher hazard of death with Gem-Nab compared with FOLFIRINOX (HR, 1.30; 95% CI, 1.06–1.59) (23).

These findings reinforce the need for individualized regimen selection in mPAC, consistent with guideline-supported evidence for both FOLFIRINOX and Gem-Nab as high-value options for eligible patients (8, 9, 11). Real-world practice and recent trial data, such as the GENERATE trial demonstrating noninferiority of Gem-Nab to modified FOLFIRINOX (34), underscore the idea that regimen selection should reflect clinical status, toxicity considerations (e.g., neuropathy and platinum intolerance), and patient preferences.

Variations in treatment duration, reflected by differences in TTD between regimens, highlight the importance of considering long-term treatment dynamics during shared decision-making (13, 19, 31). Importantly, treatment discontinuation may occur for reasons unrelated to disease progression, including toxicity, patient preference, or logistical considerations, and therefore, TTD should not be interpreted as a direct surrogate for treatment efficacy (26). Similar observations have been described in recent real-world analyses showing increased regimen diversification and evolving sequencing strategies over time (13, 19, 35). Equity-focused strategies, including improved access to academic centers and mitigation of insurance or socioeconomic-related barriers, may help to address disparities in regimen access and clinical outcomes (21, 27).

Strengths of this study include the large and diverse cohort and the use of a nationwide oncology EHR-derived database sampled from community and academic cancer centers in the US. Demographic and geographic distributions were reported as generally similar to the Surveillance, Epidemiology, and End Results Program and the National Program of Cancer Registries databases (36), and the application of standard statistical methods aligned with real-world comparative effectiveness analyses, including descriptive summaries, multinomial logistic regression for treatment selection, and Kaplan–Meier and Cox proportional hazards models for OS and TTD.

Several limitations should be acknowledged. As an observational, retrospective analysis, this study is subject to residual confounding despite adjustment for a broad range of measured clinical and sociodemographic characteristics. Socioeconomic status was assessed using area−level indicators, which may not have fully captured individual−level circumstances and may have introduced ecological bias, a recognized limitation when group−level measures are used as proxies for individual−level socioeconomic characteristics and may result in misclassification (37). Although complementary approaches, including multivariable regression, were applied to mitigate confounding, unmeasured or incompletely captured clinical factors such as baseline disease burden (e.g., history of definitive surgical resection and the anatomic distribution of metastatic lesions) and patient frailty that were not fully captured by baseline ECOG PS may have influenced treatment selection and outcomes. Accordingly, findings should be interpreted as associative rather than causal in nature. Additionally, interaction effects and nonlinear relationships were not explored, and although standard model diagnostics (e.g., assessment of multicollinearity) were performed, more comprehensive evaluation of model assumptions and complex relationships may have been limited, potentially reducing the ability to detect more complex associations in this heterogeneous real−world cohort. Although certain advanced analytic approaches were applied, broader use of methods such as propensity score approaches or time-dependent analyses may have been limited by constraints in covariate completeness, treatment timing precision, and event attribution in the EHR dataset. Certain key clinical variables were incompletely captured, including ECOG PS and metastatic sites. ECOG PS was frequently undocumented, particularly among patients who did not initiate systemic therapy, suggesting that missingness was not random but related to patient characteristics, with limited ability to fully account for baseline functional differences between treatment groups; as a result, both treatment selection and outcome analyses may be subject to residual bias despite adjustment and imputation. Furthermore, survival time was measured from metastatic diagnosis rather than treatment initiation, which may introduce immortal time bias, particularly when comparing treated and untreated cohorts. Although this approach ensured a uniform time origin, patients who survived long enough to initiate therapy may be inherently selected. Liver metastasis, an important prognostic factor in mPAC, was not accounted for in the analysis, which may have influenced observed survival differences. Documentation of breast cancer gene 1/2 (*BRCA1/2*) and partner and localizer of BRCA2 (*PALB2*) mutation status was also limited, constraining interpretation in light of recent evidence showing that platinum−containing regimens have differential benefit based on homologous-recombination deficiency (38). Moreover, nearly one-fifth of patients with ECOG PS 2+ received FOLFIRINOX, which may reflect unmeasured clinical judgment or incomplete functional status documentation. Finally, adverse event and treatment tolerability data were not available in the EHR dataset, preventing detailed assessment of toxicity, dose modifications, or treatment interruptions. Sensitivity analyses specifically evaluating the impact of missing baseline ECOG PS were not performed; therefore, residual bias related to ECOG PS missingness may remain despite adjustment and imputation.

Additional studies, including prospective studies, are needed to validate these findings and further explore determinants of treatment initiation, regimen selection, and outcomes as new therapeutic options emerge (31, 35). Additional qualitative or quantitative research may help to elucidate reasons underlying treatment noninitiation or discontinuation. Future research should focus on interventions aimed at reducing disparities in access to systemic therapy, particularly for underserved populations (27). Continued real-world evidence comparing established regimens with emerging therapies such as NALIRIFOX and biomarker-directed strategies will be critical as the therapeutic landscape evolves (13, 31, 39). Future studies with more granular capture of metastatic patterns and supportive care interventions may further refine understanding of treatment sequencing and outcomes.

## 5 Conclusions

In this cohort, treatment with FOLFIRINOX was associated with longer survival and treatment persistence than Gem-Nab. Although models were adjusted for measured baseline characteristics, these findings should be interpreted as associative given that residual confounding and potential bias related to treatment timing may remain. Approximately one-third of patients did not receive systemic anticancer treatment within 180 days of metastatic diagnosis, which, for some patients, may reflect unmet need or gaps in access to care. Treatment patterns reflected clinical and sociodemographic heterogeneity, aligning with real-world practice and previously published observational cohorts (25, 28, 29). Importantly, patient factors such as age, performance status, disease stage, socioeconomic status, and practice setting influenced regimen selection, highlighting differences in how 1L therapies are selected and applied in routine clinical practice. These findings highlight the need for targeted strategies to expand access to care, promote equitable care delivery, and support individualized treatment selection for patients with mPAC.

## Data Availability

Researchers may request access to anonymized participant-level data, trial-level data, and study protocols from Astellas-sponsored clinical trials. Details are available through the Astellas Clinical Trial Transparency website: https://www.clinicaltrials.astellas.com/transparency/.
