# Supplementary material for: Treatment patterns and outcomes of patients with a diagnosis of metastatic pancreatic adenocarcinoma in the United States, 2019–2024
Source: Front Oncol. 2026 Jul 20;16:1844261. doi: 10.3389/fonc.2026.1844261 (PMC13429506; doi:10.3389/fonc.2026.1844261)
Supplement: Supplementary file 1 [file Table1.docx]

**Supplementary materials**

**Supplementary Table S1.** Key patient characteristics by 1L therapy in metastatic pancreatic cancer**.**

| **Characteristic** | **Overall**  **(N = 9439)** | **No treatment**  **(n = 3160)** | **1L FOLFIRINOX^a^**  **(n = 2277)** | **Other 1L FU regimens**  **(n = 821)** | **1L Gem-Nab**  **(n = 2605)** | **Other 1L Gem regimens**  **(n = 465)** | **Others**  **(n = 111)** |
| --- | --- | --- | --- | --- | --- | --- | --- |
| **Age at mPAC diagnosis,**  **mean (SD)** | 69.2 (9.8) | 70.3 (10.1) | 64.8 (8.9) | 70.2 (9.6) | 70.4 (9.1) | 73.6 (9.6) | 73.0 (9.2) |
| **Age groups, n (%)** |  |  |  |  |  |  |  |
| < 55 | 749 (7.9) | 244 (7.7) | 282 (12.4) | 57 (6.9) | 144 (5.5) | 17 (3.7) | 5 (4.5) |
| 55–64 | 2126 (22.5) | 617 (19.5) | 766 (33.6) | 156 (19.0) | 506 (19.4) | 67 (14.4) | 14 (12.6) |
| 65–74 | 3492 (37.0) | 1107 (35.0) | 920 (40.4) | 307 (37.4) | 985 (37.8) | 134 (28.8) | 39 (35.1) |
| > 75 | 3072 (32.5) | 1192 (37.7) | 309 (13.6) | 301 (36.7) | 970 (37.2) | 247 (53.1) | 53 (47.7) |
| **Index year**  **(diagnosis of metastatic disease), n (%)** |  |  |  |  |  |  |  |
| 2019 | 1644 (17.4) | 529 (16.7) | 385 (16.9) | 140 (17.1) | 487 (18.7) | 91 (19.6) | 12 (10.8) |
| 2020 | 1658 (17.6) | 513 (16.2) | 374 (16.4) | 151 (18.4) | 520 (20.0) | 82 (17.6) | 18 (16.2) |
| 2021 | 1742 (18.5) | 563 (17.8) | 431 (18.9) | 142 (17.3) | 478 (18.3) | 103 (22.2) | 25 (22.5) |
| 2022 | 1778 (18.8) | 641 (20.3) | 429 (18.8) | 142 (17.3) | 463 (17.8) | 80 (17.2) | 23 (20.7) |
| 2023 | 1685 (17.9) | 622 (19.7) | 417 (18.3) | 137 (16.7) | 410 (15.7) | 72 (15.5) | 27 (24.3) |
| 2024 | 932 (9.9) | 292 (9.2) | 241 (10.6) | 109 (13.3) | 247 (9.5) | 37 (8.0) | 6 (5.4) |
| **Sex, n (%)** |  |  |  |  |  |  |  |
| Male | 4981 (52.8) | 1611 (51.0) | 1287 (56.5) | 404 (49.2) | 1394 (53.5) | 231 (49.7) | 54 (48.6) |
| Female | 4455 (47.2) | 1547 (49.0) | 990 (43.5) | 416 (50.7) | 1211 (46.5) | 234 (50.3) | 57 (51.4) |
| Unreported | 3 (0.03) | 2 (0.1) | 0 | 1 (0.1) | 0 | 0 | 0 |
| **Race and ethnicity, n (%)** |  |  |  |  |  |  |  |
| Non-Hispanic White | 4784 (50.7) | 1696 (53.7) | 1149 (50.5) | 395 (48.1) | 1275 (48.9) | 206 (44.3) | 63 (56.8) |
| Non-Hispanic Black | 768 (8.1) | 251 (7.9) | 180 (7.9) | 83 (10.1) | 211 (8.1) | 39 (8.4) | 4 (3.6) |
| Asian | 144 (1.5) | 52 (1.6) | 41 (1.8) | 15 (1.8) | 30 (1.2) | 5 (1.1) | 1 (0.9) |
| Hispanic and Latino | 561 (5.9) | 161 (5.1) | 159 (7.0) | 50 (6.1) | 152 (5.8) | 33 (7.1) | 6 (5.4) |
| Others | 433 (4.6) | 127 (4.0) | 86 (3.8) | 40 (4.9) | 150 (5.8) | 26 (5.6) | 4 (3.6) |
| Unreported | 2749 (29.1) | 873 (27.6) | 662 (29.1) | 238 (29.0) | 787 (30.2) | 156 (33.5) | 33 (29.7) |
| **History of smoking, n (%)** |  |  |  |  |  |  |  |
| Yes (Current and ex-smoker) | 4924 (52.2) | 1615 (51.1) | 1216 (53.4) | 385 (46.9) | 1414 (54.3) | 239 (51.4) | 55 (49.5) |
| No | 4458 (47.2) | 1498 (47.4) | 1059 (46.5) | 432 (52.6) | 1189 (45.6) | 224 (48.2) | 56 (50.5) |
| Unreported | 57 (0.6) | 47 (1.5) | 2 (0.1) | 4 (0.5) | 2 (0.1) | 2 (0.4) | 0 |
| **Insurance coverage, n (%)^b^** |  |  |  |  |  |  |  |
| Commercial health plan | 3934 (41.7) | 1158 (36.6) | 1134 (49.8) | 368 (44.8) | 1068 (41.0) | 165 (35.5) | 41 (36.9) |
| Medicare | 4077 (43.2) | 1437 (45.5) | 766 (33.6) | 358 (43.6) | 1213 (46.6) | 245 (52.7) | 58 (52.3) |
| Medicaid | 348 (3.7) | 107 (3.4) | 107 (4.7) | 27 (3.3) | 99 (3.8) | 7 (1.5) | 1 (0.9) |
| Other | 396 (4.2) | 106 (3.4) | 108 (4.7) | 40 (4.9) | 111 (4.3) | 25 (5.4) | 6 (5.4) |
| Self-pay | 684 (7.2) | 352 (11.1) | 162 (7.1) | 28 (3.4) | 114 (4.4) | 23 (4.9) | 5 (4.5) |
| **Practice setting, n (%)** |  |  |  |  |  |  |  |
| Academic | 2774 (29.4) | 1946 (61.6) | 1653 (72.6) | 613 (74.7) | 2031 (78.0) | 340 (73.1) | 82 (73.9) |
| Community | 6665 (70.6) | 1214 (38.4) | 624 (27.4) | 208 (25.3) | 574 (22.0) | 125 (26.9) | 29 (26.1) |
| **Socioeconomic status, n (%)** |  |  |  |  |  |  |  |
| Quintile 1 (lowest) | 1311 (13.9) | 420 (13.3) | 316 (13.9) | 103 (12.5) | 388 (14.9) | 68 (14.6) | 16 (14.4) |
| Quintile 2 | 1541 (16.3) | 514 (16.3) | 342 (15.0) | 130 (15.8) | 461 (17.7) | 79 (17.0) | 15 (13.5) |
| Quintile 3 | 1789 (19.0) | 623 (19.7) | 449 (19.7) | 155 (18.9) | 484 (18.6) | 65 (14.0) | 13 (11.7) |
| Quintile 4 | 2094 (22.2) | 673 (21.3) | 518 (22.7) | 194 (23.6) | 573 (22.0) | 105 (22.6) | 31 (27.9) |
| Quintile 5 (highest) | 1884 (20.0) | 657 (20.8) | 450 (19.8) | 173 (21.1) | 476 (18.3) | 100 (21.5) | 28 (25.2) |
| Unreported | 820 (8.7) | 273 (8.6) | 202 (8.9) | 66 (8.0) | 223 (8.6) | 48 (10.3) | 8 (7.2) |
| **Baseline ECOG PS, n (%)** |  |  |  |  |  |  |  |
| 0/1 | 4779 (50.6) | 184 (5.8) | 1800 (79.1) | 580 (70.6) | 1877 (72.1) | 263 (56.6) | 75 (67.6) |
| 2/3/4 | 1006 (10.7) | 49 (1.6) | 180 (7.9) | 142 (17.3) | 454 (17.4) | 157 (33.8) | 24 (21.6) |
| Unreported | 3654 (38.7) | 2927 (92.6) | 297 (13.0) | 99 (12.1) | 274 (10.5) | 45 (9.7) | 12 (10.8) |
| **Disease staging at initial presentation, n (%)** |  |  |  |  |  |  |  |
| Early stage/locally advanced | 2593 (27.5) | 923 (29.2) | 298 (13.1) | 394 (48.0) | 796 (30.6) | 130 (28.0) | 52 (46.8) |
| De novo metastatic disease | 5991 (63.5) | 1859 (58.8) | 1887 (82.9) | 326 (39.7) | 1586 (60.9) | 294 (63.2) | 39 (35.1) |
| Unreported | 855 (9.1) | 378 (12.0) | 92 (4.0) | 101 (12.3) | 223 (8.6) | 41 (8.8) | 20 (18.0) |
| **Sites of metastasis, n (%)^b^** |  |  |  |  |  |  |  |
| Liver | 1628 (17.2) | 493 (15.6) | 475 (20.9) | 130 (15.8) | 434 (16.7) | 82 (17.6) | 14 (12.6) |
| Peritoneum | 290 (3.1) | 98 (3.1) | 59 (2.6) | 34 (4.1) | 77 (3.0) | 21 (4.5) | 1 (0.9) |
| Lung | 259 (2.7) | 75 (2.4) | 53 (2.3) | 26 (3.2) | 73 (2.8) | 24 (5.2) | 8 (7.2) |
| Brain | 12 (0.1) | 8 (0.3) | 2 (0.1) | 0 | 1 (< 1) | 0 | 1 (0.9) |
| Bone | 119 (1.3) | 36 (1.1) | 28 (1.2) | 14 (1.7) | 36 (1.4) | 4 (0.9) | 1 (0.9) |
| Others | 531 (5.6) | 171 (5.4) | 124 (5.4) | 56 (6.8) | 147 (5.6) | 26 (5.6) | 7 (6.3) |
| Unknown | 7172 (76.0) | 2478 (78.4) | 1667 (73.2) | 614 (74.8) | 1976 (75.9) | 353 (75.9) | 84 (75.7) |
| **Germline *BRCA1* mutation status, n (%)** |  |  |  |  |  |  |  |
| Wild-type | 1280 (13.6) | 381 (12.1) | 194 (8.5) | 204 (24.8) | 399 (15.3) | 63 (13.5) | 39 (35.1) |
| Mutated | 26 (0.3) | 2 (0.1) | 11 (0.5) | 6 (0.7) | 2 (0.1) | 2 (0.4) | 3 (2.7) |
| Unreported | 8133 (86.2) | 2777 (87.9) | 2072 (91.0) | 611 (74.4) | 2204 (84.6) | 400 (86.0) | 69 (62.2) |
| **Germline *BRCA2* mutation status, n (%)** |  |  |  |  |  |  |  |
| Wild-type | 1264 (13.4) | 375 (11.9) | 198 (8.7) | 203 (24.7) | 394 (15.1) | 60 (12.9) | 34 (30.6) |
| Mutated | 42 (0.4) | 8 (0.3) | 7 (0.3) | 7 (0.9) | 7 (0.3) | 5 (1.1) | 8 (7.2) |
| Unreported | 8133 (86.2) | 2777 (87.9) | 2072 (91.0) | 611 (74.4) | 2204 (84.6) | 400 (86.0) | 69 (62.2) |
| **Somatic *BRCA1* mutation**  **status, n (%)** |  |  |  |  |  |  |  |
| Wild-type | 717 (7.6) | 236 (7.5) | 79 (3.5) | 110 (13.4) | 219 (8.4) | 48 (10.3) | 25 (22.5) |
| Mutated | 13 (0.1) | 2 (0.1) | 4 (0.2) | 2 (0.2) | 1 (< 1) | 3 (0.6) | 1 (0.9) |
| Unreported | 8709 (92.3) | 2922 (92.5) | 2194 (96.4) | 709 (86.4) | 2385 (91.6) | 414 (89.0) | 85 (76.6) |
| **Somatic *BRCA2* mutation**  **status, n (%)** |  |  |  |  |  |  |  |
| Wild-type | 707 (7.5) | 234 (7.4) | 78 (3.4) | 112 (13.6) | 215 (8.3) | 44 (9.5) | 24 (21.6) |
| Mutated | 23 (0.2) | 4 (0.1) | 5 (0.2) | 0 | 5 (0.2) | 7 (1.5) | 2 (1.8) |
| Unreported | 8709 (92.3) | 2922 (92.5) | 2194 (96.4) | 709 (86.4) | 2385 (91.6) | 414 (89.0) | 85 (76.6) |
| **Baseline comorbidities, n (%)^b^** |  |  |  |  |  |  |  |
| Type 2 diabetes mellitus | 992 (10.5) | 341 (10.8) | 226 (9.9) | 98 (11.9) | 263 (10.1) | 53 (11.4) | 11 (9.9) |
| Hypertension | 1396 (14.8) | 462 (14.6) | 338 (14.8) | 130 (15.8) | 386 (14.8) | 65 (14.0) | 15 (13.5) |
| Coronary heart disease | 38 (0.4) | 19 (0.6) | 6 (0.3) | 3 (0.4) | 8 (0.3) | 2 (0.4) | 0 |
| Heart failure | 123 (1.3) | 53 (1.7) | 16 (0.7) | 15 (1.8) | 32 (1.2) | 7 (1.5) | 0 |
| Liver disease | 707 (7.5) | 265 (8.4) | 157 (6.9) | 65 (7.9) | 168 (6.4) | 43 (9.2) | 9 (8.1) |
| Renal disease | 207 (2.2) | 98 (3.1) | 15 (0.7) | 22 (2.7) | 60 (2.3) | 10 (2.2) | 2 (1.8) |
| Immunocompromised conditions | 51 (0.5) | 16 (0.5) | 10 (0.4) | 7 (0.9) | 15 (0.6) | 1 (0.2) | 2 (1.8) |
| Hepatitis B/C infection | 17 (0.2) | 6 (0.2) | 6 (0.3) | 1 (0.1) | 3 (0.1) | 1 (0.2) | 0 |
| None of the above | 7210 (76.4) | 2401 (76.0) | 1738 (76.3) | 622 (75.8) | 2017 (77.4) | 348 (74.8) | 84 (75.7) |

^a^ Regimen included modified FOLFIRINOX.  ^b^ Patients may have belonged to ≥ 1 category.
1L, first-line; *BRCA*, breast cancer gene; ECOG PS, Eastern Cooperative Oncology Group performance status; FOLFIRINOX, folinic acid (leucovorin), FU, irinotecan, oxaliplatin; FU, fluorouracil; Gem, gemcitabine; Gem-Nab, gemcitabine plus nab-paclitaxel; SD, standard deviation.
